# Comparative analysis of the influence of BpfA and BpfG on biofilm development and current density in *Shewanella oneidensis* under oxic, fumarate- and anode-respiring conditions

**DOI:** 10.1038/s41598-024-73474-w

**Published:** 2024-10-05

**Authors:** Edina Marlen Klein, Hannah Heintz, René Wurst, Simon Schuldt, Hendrik Hähl, Karin Jacobs, Johannes Gescher

**Affiliations:** 1 Institute of Technical Microbiology, University of Technology Hamburg, 21073 Hamburg, Germany; 2 Experimental Physics, Center for Biophysics, Saarland University, 66123 Saarbrücken, Germany; 3 Max Planck School Matter to Life, 69120 Heidelberg, Germany

**Keywords:** Applied microbiology, Biofilms, Genetic engineering, Lab-on-a-chip, Atomic force microscopy, Extracellular matrix

## Abstract

Biofilm formation by *Shewanella oneidensis* has been extensively studied under oxic conditions; however, relatively little is known about biofilm formation under anoxic conditions and how biofilm architecture and composition can positively influence current generation in bioelectrochemical systems. In this study, we utilized a recently developed microfluidic biofilm analysis setup with automated 3D imaging to investigate the effects of extracellular electron acceptors and synthetic modifications to the extracellular polymeric matrix on biofilm formation. Our results with the wild type strain demonstrate robust biofilm formation even under anoxic conditions when fumarate is used as the electron acceptor. However, this pattern shifts when a graphite electrode is employed as the electron acceptor, resulting in biofilm formation falling below the detection limit of the optical coherence tomography imaging system. To manipulate biofilm formation, we aimed to express BpfG with a single amino acid substitution in the catalytic center (C116S) and to overexpress *bpfA*. Our analyses indicate that, under oxic conditions, overarching mechanisms predominantly influence biofilm development, rather than the specific mutations we investigated. Under anoxic conditions, the *bpfG* mutation led to a quantitative increase in biofilm formation, but both strains exhibited significant qualitative changes in biofilm architecture compared to the controls. When an anode was used as the sole electron acceptor, both the *bpfA* and *bpfG* mutations positively impacted mean current density, yielding a 1.8-fold increase for each mutation.

**Supplementary Information:**

The online version contains supplementary material available at 10.1038/s41598-024-73474-w.

## Introduction

*Shewanella oneidensis* MR1 is the best understood model organism regarding dissimilatory metal reduction and extracellular electron transfer onto carbon electrodes in bioelectrochemical systems (BES)^1^. However, strains of *S. oneidensis* generally exhibit lower rates of dissimilatory iron reduction and lower current densities compared to the other prominent model organism, *Geobacter sulfurreducens*^2^. The prevailing view is that *G. sulfurreducens* has evolved as a specialist for anaerobic respiration with insoluble electron acceptors, while *S. oneidensis* is as a respiration generalist, which is corroborated by its ability of respiring with the widest range of electron acceptors (including oxygen) known to date. A comparison of the biochemical machinery involved in extracellular electron transfer reveals that the overall strategies for transporting electrons to the cell surface are quite similar in both *S. oneidensis* and *G. sulfurreducens*^3–5^. Both organisms have developed an electron conduit through the outer membrane, consisting of one *c*-type cytochrome on the periplasmic side and one or two *c-*type cytochromes on the extracellular side of the outer membrane. The primary difference in their electron transfer strategies lies in the extension of the electron transfer chain beyond the cell surface in *G. sulfurreducens*^6–9^. While the mechanisms for electron transfer from the cytoplasmic membrane through the periplasm and the outer membrane are similar, *G. sulfurreducens* has developed the ability to conductively connect cells with one another and with electrodes using nanowires^8,10–14^. This capability has led to the hypothesis that the formation of multilayered, thick biofilms under anoxic conditions on solid electron acceptors is directly related to the organism’s ability to generate conductive extracellular polymeric substances (EPS). Although *S. oneidensis* does not produce similar nanowires, cell-to-cell electron transfer could still occur through direct electron exchange between outer membrane-exposed cytochromes or potentially with the aid of outer membrane vesicles produced by *S. oneidensis*, which were previously thought to function as conductive protein nanowires^15–20^.

Biofilm formation by *S. oneidensis* has been well studied under oxic conditions^21–25^; however, mechanisms that trigger biofilm formation under anoxic conditions and the influence of biofilm architecture and composition on current generation are not well understood. Recently, Edel et al.^25^. examined the formation of *S. oneidensis* biofilms on anode surfaces, demonstrating that riboflavin can act similarly to a quorum-sensing molecule in this γ-proteobacterium. The enhanced biofilm formation resulting from riboflavin addition led to increased current densities. A limited number of candidate proteins showed concentration changes influenced by this quorum sensing mechanism, including the UDP-N-acetylglucosamine C4 epimerase WbpP. This observation supports the hypothesis that sugar chemistry within the EPS can modify biofilm characteristics, promoting greater biofilm growth under anoxic conditions and consequently higher current densities.

A biofilm formation mechanism mediated by an extracellular protein network is the Bpf system, which is present in *S. oneidensis* as well as other organisms. This system employs a type I secretion system to export a protein called BpfA (biofilm-promoting factor) from the cell^26^. BpfA acts as an outer membrane-bound cell connector, and its activity can be modulated by the periplasmic protease BpfG, which is essential for BpfA export and can cleave BpfA, leading to its release and biofilm dispersal. The regulatory process likely involves cellular cyclic di-guanosine monophosphate (c-di-GMP) concentrations and the cytoplasmic membrane-bound protein BpfD, which interacts with BpfG, possibly through its periplasmic domain. Notably, previous experiments demonstrated that overproduction of BpfG in *S. oneidensis* can significantly enhance biofilm formation under oxic conditions^26^.

Given the critical roles of BpfA and BpfG in biofilm formation observed in these studies, we hypothesized that these factors would also be significant under anoxic respiratory conditions. Therefore, we aimed to systematically investigate and compare the influences of BpfA and BpfG on *S. oneidensis* biofilm formation under various conditions: with oxygen, fumarate, or an anode as the sole terminal electron acceptor under continuous flow conditions. Our objective was to determine whether this system could be manipulated to steer biofilm production and whether such steering could be applied to influence current generation in bioelectrochemical systems.

In this study, we utilized a recently developed microfluidic biofilm analysis setup with automated 3D imaging^27^ to assess the effects of these biofilm-altering protein components. A mutant producing BpfG with a point mutation in the catalytic center of the protease part (C116S), as well as a strain overexpressing *bpfA* were investigated. The analyses reveal that under oxic conditions, superordinate mechanisms rather than the investigated mutations take effect and determine biofilm development. Under anoxic conditions, however, only the *bpfG* mutation resulted in a quantitative increase in biofilm formation, while the qualitative architecture of the biofilms was markedly altered across all strains compared to the controls. When an anode was used as the sole electron acceptor, both *bpfA* and *bpfG* mutations resulted in positive effects, each contributing to a 1.8-fold increase in mean current density.

## Materials and methods

### Media and growth condition

All strains used in this study are listed in Table S1. *S. oneidensis* cells were pre-cultured overnight at 30 °C and 160 rpm in Lysogeny-Broth medium (LB)^28^ and then transferred to minimal medium. This M4 medium^29^ contained 1.26 mM K_2_HPO_4_, 0.66 mM KH_2_PO_4_, 4.78 mM HEPES, 2.02 mM NaHCO_3_, 9.01 mM NH_4_SO_4_, 150.07 mM NaCl, 1 g l^− 1^ casein hydrolysate, 1 mM MgSO_4_, 0.1 mM CaCl_2_ and 10 ml trace elements (5 µM CoCl_2_, 0.2 µM CuSO_4_, 57 µM H_3_BO_3_, 5.4 µM FeCl_2_, 1.3 µM MnSO_4_, 67.2 µM Na_2_EDTA, 3.9 µM Na_2_MoO_4_, 1.5 µM Na_2_SeO_4_, 5 µM NiCl_2_, and 1 µM ZnSO_4_). The pH of the medium was adjusted to 7.4. Oxic experiments were conducted in M4 medium containing 70 mM lactate and anoxic experiments were performed in anoxic M4 medium with 70 mM lactate as carbon and electron source and 100 mM fumarate as electron acceptor. In order to produce anoxic media, the bottles were sealed with rubber stoppers and the headspace of the bottles was replaced with nitrogen by alternating between vacuum and nitrogen overpressure on a repeated basis. Pre-cultures for bioelectrochemical experiments were grown in anoxic M4 medium with 70 mM lactate and 100 mM fumarate. Bioelectrochemical experiments were performed in medium containing 70 mM lactate and no electron acceptor except for the anode of the system. All precultures were grown overnight at 30 °C in M4 minimal medium, with oxic cultures incubated at 160 rpm. Prior to inoculation, cells were harvested by centrifugation (5 min, 6,000 g) and washed twice with medium containing neither electron donor nor electron acceptor. The cells were then adjusted to a suitable OD_600_ as indicated below.

### Construction of marker-less mutants

Marker-less genomic alterations in *S. oneidensis* were conducted using the suicide vector pMQ150^26,30^. The vector was linearized with BamHI and SalI. The *bpfG* gene and the promoter region of *bpfA* were initially deleted, after which modified variants were reintegrated into the genome. Fragments measuring 500 bp upstream and downstream of the respective genes were amplified using primers 1–4 for the *bpfG* deletion and primers 5–8 for the P_*bpfA*_ deletion. (Table S2). The fragments contained an overlap to the pMQ150 plasmid as well as to each other. Cloning of the fragments in the pMQ plasmid was performed according to Gibson and colleagues^31^ and the resulting plasmids were transformed into the *E. coli* mating strain WM3064. The suicide vector was then conjugated into *S. oneidensis* and all further steps were conducted as described by Saltikov and Newman^32^. After successful deletion, the point mutation C116S was introduced to *bpfG* by using primers 9–10. The promoter region of *cymA* was amplified with primers 12 and 14 and the 500 bp upstream and downstream regions with primers 5 and 11 as well as 8 and 13, respectively. The remaining procedure was carried out in the same way as described above which led to a strain with the replacement of the natural promoter with the *cymA* promoter and a strain with a point mutation in *bpfG*. All modifications were verified by PCR analysis (primers 15 to 22) and subsequent sequencing.

### Microfluidic biofilm cultivation platform

Biofilms were studied using a newly developed microfluidic cultivation platform operating under laminar flow conditions^27^. A straight channel design was chosen for the reactors, and all experiments were performed in incubation chambers consisting of two compartments, with one chamber always holding a triplicate. We employed the same microfluidic reactor geometry as described in Klein et al.^27^. with the exception that the void space in the PDMS cover used for electrode insertion was also filled with PDMS. All experiments were conducted as described previously^27^.

For oxic and anoxic biofilm experiments, the cells were adjusted to a final OD_600_ of 0.2. For inoculation, the cells were pumped into the reactors through a side port with 2 ml h^−1^, and 2 ml h^−1^ medium was added simultaneously. Anoxic conditions were ensured by covering the chamber with a polycarbonate lid and continuously flushing the chamber headspace with 30 ml min^−1^ N_2_ gas. After two hours of inoculation, the side port was closed, and the reactors were supplied with medium at a flow rate of 4 ml h^−1^ for the remainder of the experiment. The microfluidic reactors were set up with the glass side down during cultivation.

For the bioelectrochemical experiments, graphite composite electrodes (PPG 86, Eisenhuth GmbH & Co. KG, Osterode am Harz, Germany) with an effective surface area of 3 × 10 mm were integrated into the cultivation channel, as described^27^. Two reactors were connected in series to form a complete BES. The upstream reactor contained the working electrode and carried a customized Ag/AgCl reference electrode behind the working electrode. The cathode compartment was operated under oxic conditions, while the anode compartment was kept oxygen-free. The electrodes were connected to a potentiostat (BioLogic VMP-300, Seyssinet-Pariset, France) via a silver foil (0.1 mm; Chempur, Karlsruhe, Germany). The anode potential was set to 0 V (vs. standard hydrogen electrode (SHE)) and the current was monitored for 48 h. Inoculation was performed similarly to the oxic and anoxic biofilm experiments with a final OD_600_ of 2.0. During cultivation, the reactors were placed with the glass side up and the electrode at the bottom.

### Optical coherence tomography (OCT)

Optical coherence tomography (OCT) was conducted according to Klein and colleagues^27^. The OCT probing head was mounted on a gantry robot (DLE-RG-0003, igus^®^ GmbH, Cologne, Germany) for semiautonomous image acquisition^27^. This way, development of the biofilm was monitored throughout the experiment with high reproducibility and low workload. OCT images were acquired with a GanymedeTM spectral domain system (GAN611C1-SP1, Thorlabs GmbH, Dachau, Germany) and analyzed as recently described^27,33^. Data sets were processed using (Fiji Is Just) ImageJ version 2.1.0/1.53^34^. Height maps of the anodes were generated based on the procedure established by Wagner and Horn^35^, and the surface coverage was assessed based on these height maps. The porosity of the biofilms was determined by correlating the actual height with a theoretically calculated height generated from the biovolume (100% density) using the following formula:

1 $$\Phi =\frac{{{h_{real}} - {h_{theoretical}}}}{{{h_{real}}}} \times 100$$

In order to automate data acquisition and processing as far as possible and to counteract the imperfect repeatability in x-y-positioning of the gantry robot, the OCT images were larger (as seen from above) than the actual cultivation channel. Consequently, parts of the image had to be cropped to be excluded from the calculations. To automate this step, only 50% of the respective cultivation channel was analyzed. Thus, the lateral walls were not included in the calculation of biovolume, biofilm height, surface coverage and porosity. Only the height maps show the entire channel.

### Preparation of cells for single-cell force spectroscopy

The *S. oneidensis* variants were cultured as follows: One bacterial colony of each strain was added to 5 mL of LB medium (LB-Medium (Lennox), Carl Roth, Karlsruhe, Germany) and incubated overnight at 30 °C and 150 rpm for 16 h. To ensure that only bacteria from the exponential phase are used, another 40 µL of the overnight solution were cultivated in 4 mL LB-Medium for 2.5 h at 30 °C and 150 rpm, resulting in an optical density of ~ 0.05 at 600 nm. Afterwards the bacterial solution was washed three times with phosphate-buffered saline (PBS, pH 7.3) (Tablets, Sigma-Aldrich Merck KGaA, Darmstadt, Germany) and centrifuged for 3 min at 17,000 g.

### Substrate preparation

For the adhesion measurements, hydrophobized silicon wafers were used as test surfaces. To fabricate these surfaces, the wafers were cleaned and coated with a monolayer of OTS (octadecyltrichlorosilane) (CAS 112-04-9, abcr GmbH, Karlsruhe, Germany), following a protocol by Lessel et al.^36^. Before the surfaces were used in the experiments, they were initially cleaned in ethanol (99,8% VWR International, Radnor, PA, USA) for a period of five minutes, followed by another five minutes in acetone (99,8% VWR International, Radnor, PA, USA), both within an ultrasonic bath. After each cleaning step, the surfaces were dried with nitrogen. Throughout the measurement, the samples were covered with PBS.

### Single-cell force spectroscopy (SCFS) and analysis of the force-distance curves

Bacterial probes were prepared as described by Thewes et al.^37^ with the addition that the rod-shaped *Shewanella oneidensis* cells were always aligned horizontally on the tipless cantilever (MLCT-O10, Bruker-Nano, Santa Barbara, USA). The probes were always kept in humid environment to prevent bacterial desiccation. During the measurements, OTS surface and probe were constantly covered with PBS and maintained at room temperature. As previously described, the cantilever was calibrated before each measurement using the Sader method^38^. The adhesion measurements and the subsequent recording of the force-distance curves were performed on a Nanowizard 4 (Bruker Nano GmbH, Berlin, Germany). All force-distance curves were recorded with identical parameters for hydrophobic surfaces (800 nm ramp size, 800 nm/s retraction velocity of, 5 s attachment time and a force trigger of 300 pN) as described by Maikranz et al.^39^.

A series of 96 force-distance curves was then recorded for each bacterium on a square area of 100 μm² on the OTS surfaces. The data were analyzed using the JPKSPM Data Processing software, Version 8.0.117. All recorded curves were subject to the same analysis procedure: the baseline was subtracted, and the adhesion force was identified as the minimum value observed in the retraction curve^40^. Prior to the determination of the adhesion force, it was established that an adhesion event would be evaluated exclusively from a force with an absolute value of at least 50 pN, as a distinction from the baseline noise could no longer be guaranteed with a lower force.

## Results and discussion

### Under oxic conditions, superordinate mechanisms take effect and determine biofilm development

**Fig. 1:**
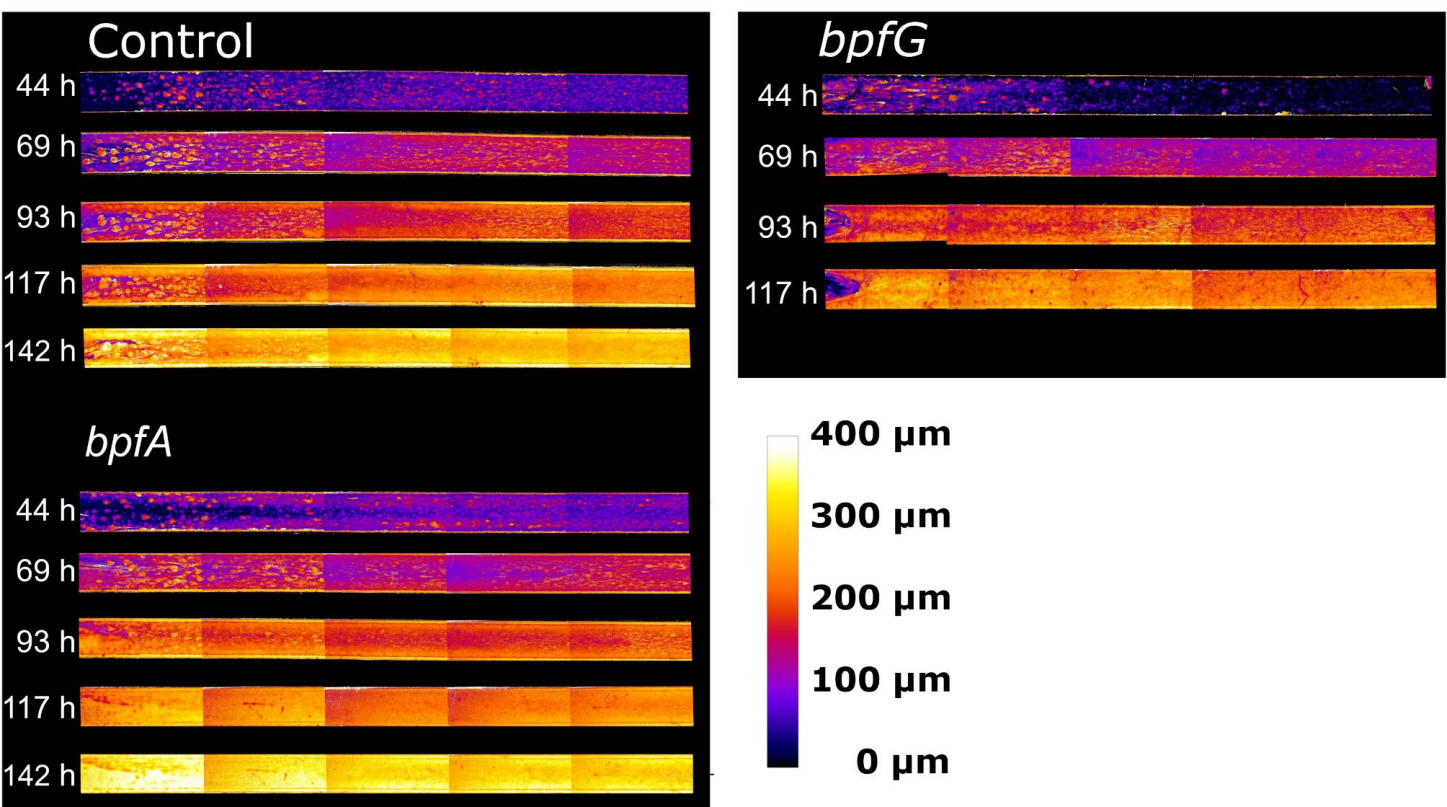
Exemplary height maps of biofilms that were cultivated using microfluidic continuous flow reactors with lactate as electron donor. Cultivation was carried out under oxic conditions and the retention time was 5.23 min at a flow rate of 4 mL h^−1^. After 44, 69, 93, 117 and 142 h five images were taken of each cultivation channel, converted into color-coded height maps by processing and combined into one image by stitching. The cultivation channels shown have a size of 3 × 45 mm and correspond to a top view of the biofilm, with a flow direction from left to right. Cultivations were performed in triplicate. Note that after 117 h, only one flow cell of *bpfA* remained due to biofilm washout, and only one flow cell of *bpfG* remained at 117 h and none at 142 h. Remaining height maps are available in Figure S3 of the supplementary materials.

The influence of an extracellular protein network on biofilm formation was systematically investigated. Central to this system is BpfA, which most likely functions as a cell-surface-attached extracellular adhesin^26^. The overexpression of *bpfA* could not be achieved using an expression plasmid, as the particularly large gene contains a high number of sequence repeats. Therefore, the native promoter was substituted with the *cymA* promoter. This promoter was selected as CymA expression was not only shown under anoxic but also under oxic and under microoxic conditions^41,42^. BpfA is part of an interactive system alongside BpfG and BpfD^26^. BpfG is a bifunctional protein that is required for both the secretion and subsequent cleavage of BpfA. Therefore, both the absence and excessive production of BpfG negatively impact biofilm formation. However, Zhou and colleagues^26^ could show that the overexpression of the BpfG (C116S) mutant results in biofilm overproduction. This point mutation occurs at the catalytic center of the protease domain, preventing cleavage of BpfA while not affecting its transport. Thus, alongside BpfA (P_cymA_), BpfG (C116S) was investigated as another potential component influencing biofilm dynamics. To systematically investigate the influence of *bpfG* (C116S) as well as overexpression of *bpfA* on biofilm formation, biofilms were developed first with lactate as electron donor under oxic conditions in a microfluidic flow-through system. The development of the biofilm was monitored with an integrated automated OCT device. Figure 1 shows examples of height maps of the biofilms over time as color-coded maps. This top view of the biofilm shows that the biofilm architecture of the strains investigated hardly differs from that of the control strains. All strains show a relatively long lag phase of almost two days. Subsequently, all biofilms grow quite uniformly without the formation of tower-like structures. However, the *bpfA* overexpressing biofilm was flushed out in a sloughing event after 93 h in two of three flow cells and only one specimen could be cultivated for the entire duration of the experiment. The entire biofilm of the *bpfG* mutant was washed away after 93 h in one replicate and after 117 h in the other two.

**Fig. 2:**
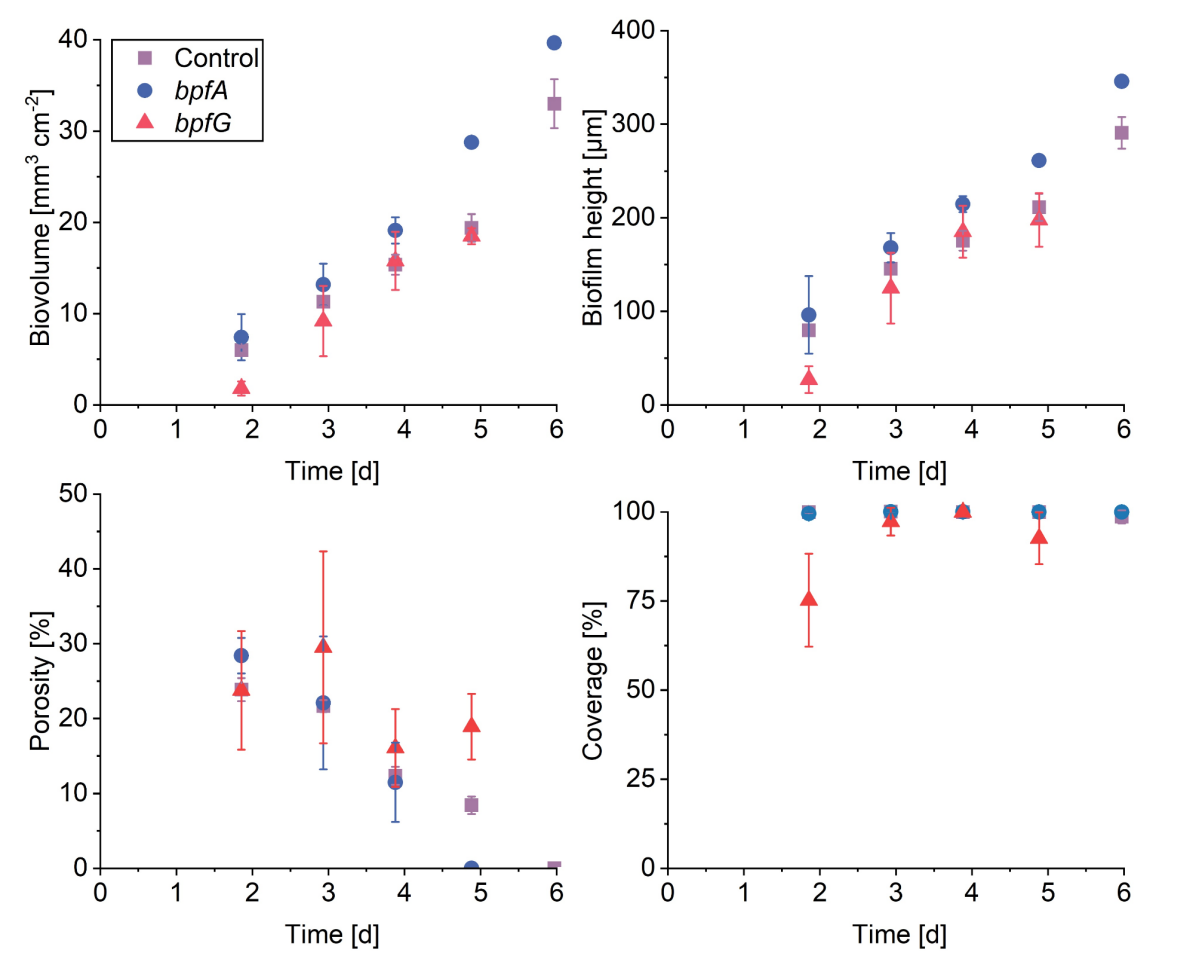
Impact of *bpfG*(C116S) as well as overexpression of *bpfA* on oxic biofilm formation. Cultivation was conducted in microfluidic flow cells under oxic conditions with lactate as electron donor and atmospheric oxygen as electron acceptor. Biovolume, biofilm height, porosity and coverage are plotted over time. Error bars represent the standard deviation from individual replicates (*n* = 3). It should be noted that after day 4 only one flow cell of the *bpfA*-expressing and two of *bpfG-*expressing strains remained after sloughing events. After day 5 only one flow cell of the triplicate for the *bpfA*-expressing strain still contained detectable biofilm. Tables S5–7 in the supportings contain the mean values and standard deviations of this figure.

This qualitative impression of similar biofilm architectures is also reflected in the quantitative data (Fig. 2). However, only the first three time points should be considered, as afterwards it was not possible anymore to collect triplicate data due to the mentioned sloughing events. The *bpfG* mutant showed significantly less biofilm in terms of biovolume and height after 44 h, but after 69 and 93 h the development was comparable to the control. In contrast, the *bpfA* mutant exhibited a similar amount of biofilm after 44 and 69 h compared to the control, however, after 93 h, the biovolume and height slightly increased reaching 19 mm^3^ cm^−2^ and 215 μm in height. The biofilms of the *bpfA* and *bpfG* mutants displayed comparable porosity to those of the control during the first four days of cultivation, though the standard deviation for the two mutants was higher than that of the control. In summary, the two mutations appear to result in slightly more unstable biofilms under the tested conditions. Notably, all strains achieved complete surface coverage after 44 h, except for *bpfG* which reached complete coverage after 69 h. While differences in biofilm development under oxic conditions were observed at certain time points, no significant fundamental differences were detected. Therefore, it was assumed that under oxic conditions superordinate mechanisms take effect and determine biofilm development. Consequently, all strains were subsequently examined under anoxic conditions to determine whether any major qualitative or quantitative differences in biofilm development would manifest.

### BpfG activity and*bpfA* expression affect anaerobic biofilm growth.

**Fig. 3:**
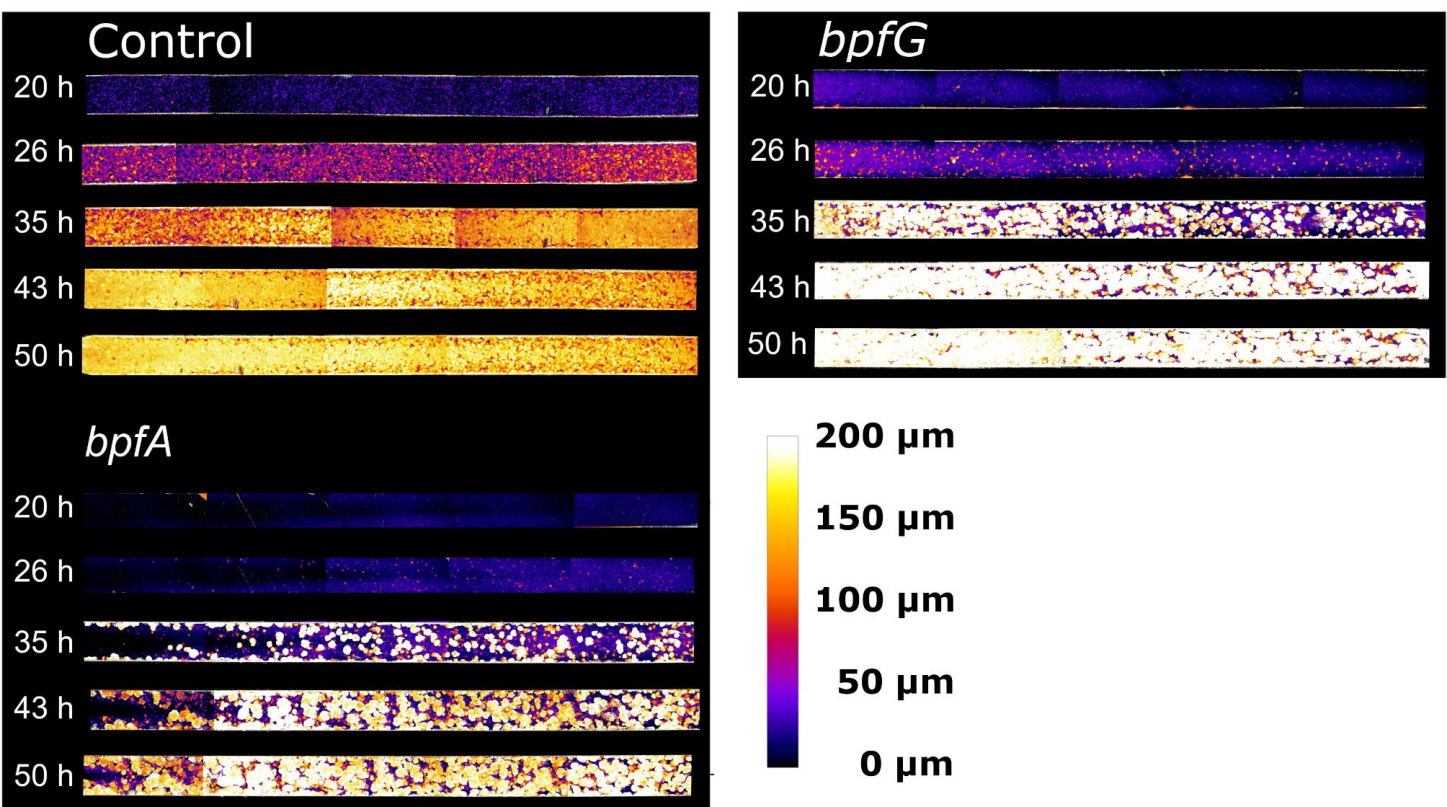
Exemplary height maps of biofilms that were cultivated using microfluidic continuous flow reactors with lactate as electron donor und fumarate as electron acceptor. Cultivation was carried out under anoxic conditions and the retention time was 5.23 min at a flow rate of 4 mL h^−1^. After 20, 26, 35, 42 and 50 h five images were taken of each cultivation channel, converted into color-coded height maps by processing and combined into one image by stitching. The cultivation channels shown have a size of 3 × 45 mm and correspond to a top view of the biofilm, with a flow direction from left to right. Cultivations were carried out in triplicate and the remaining height maps can be found in Figure S4 in the supplements.

Anoxic biofilm development was investigated using lactate as the electron donor and fumarate as the electron acceptor, while maintaining the same cultivation conditions as before. Surprisingly, the first OCT data showing visible biofilm growth were collected after just 20 h and the overall growth rate was significantly faster compared to cultivation with oxygen as the terminal electron acceptor. This increased growth rate is not unexpected, given that the solubility of oxygen from ambient air at 30 °C is only 8.05 mg/L (approximately 252 µM), whereas 100 mM fumarate was dissolved as the electron acceptor in the anoxic experiments. From the height maps of the anoxic biofilms (Fig. 3), it is clear that the biofilm architecture of the *bpfA* and *bpfG* mutants differs markedly from that of the control strain. The control strain exhibited predominantly uniform growth, whereby the biofilm consisted of numerous small colonies. In contrast, the *bpfA* and *bpfG* mutants displayed sparse colonization of the surface at the first two measurement time points, with scattered colonies approximately 50 μm in height on a uniform biofilm lawn of about 20 μm. Subsequently, the biofilms grew at a significantly increased rate and both mutants appeared to form broad tower-like structures with heights reaching over 200 μm.

**Fig. 4:**
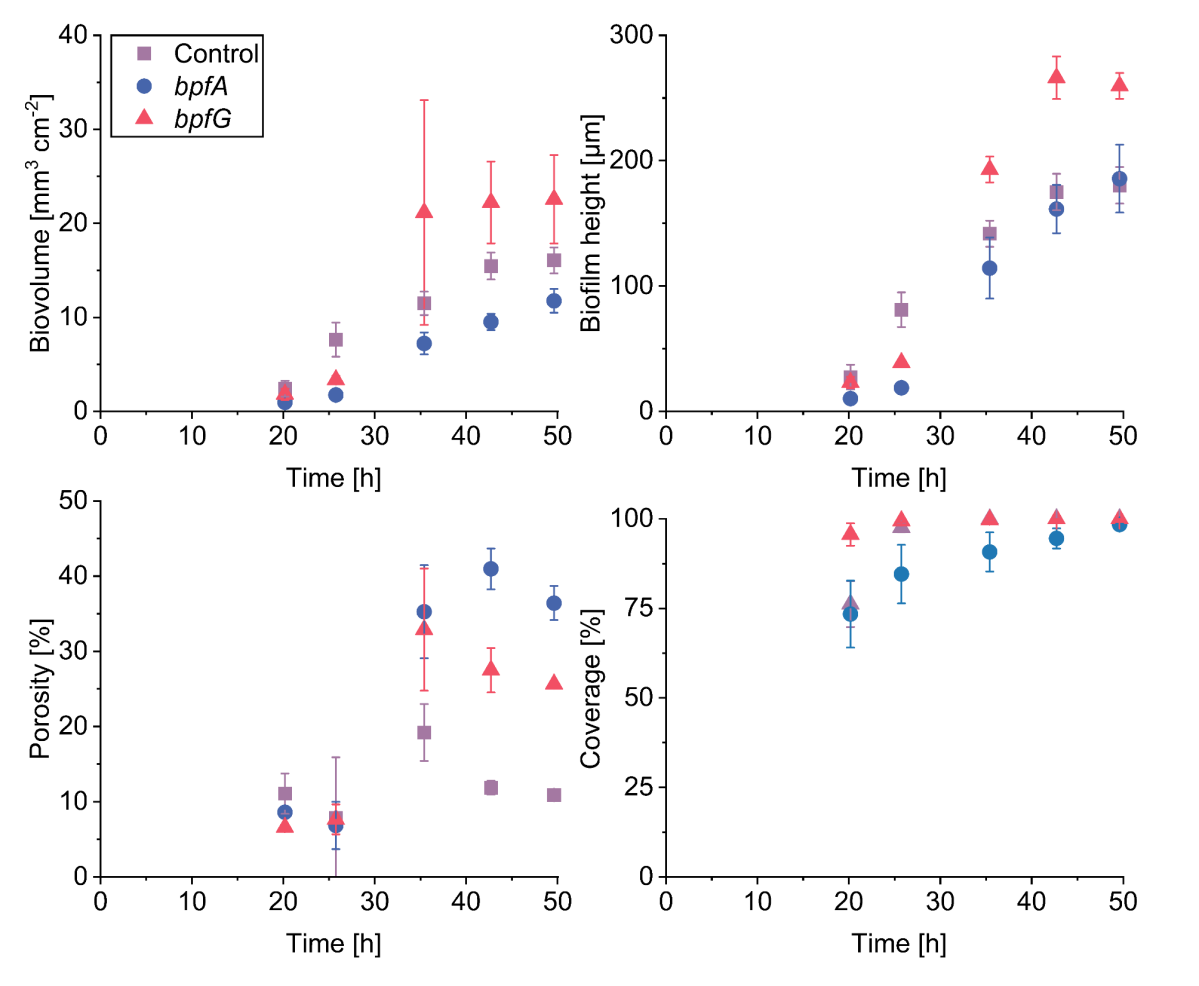
Impact of *bpfG*(C116S) as well as overexpression of *bpfA* on anoxic biofilm formation. Cultivation was conducted in microfluidic flow cells under anoxic conditions with lactate as electron donor and fumarate as electron acceptor. Biovolume, biofilm height, porosity and coverage are plotted over time. Error bars represent the standard deviation from individual replicates (*n* = 3). Tables S8–10 in the supportings contain the mean values and standard deviations of this figure.

The quantitative data (Fig. 4) support these qualitative observations (Fig. 3). In terms of biovolume and biofilm height, only the *bpfG* mutant displayed an increase compared to the control strain. Notably, the biovolume of the *bpfA* mutant was significantly lower than that of the control strain after 35 h. All strains achieved complete surface coverage after a maximum of 35 h, except for the *bpfA* mutant, which required 50 h to reach complete coverage. However, both mutants demonstrated a significant increase in biofilm porosity after 26 h of cultivation.

### Activating the Bpf system leads to increased current density in bioelectrochemical continuous flow reactors

**Fig. 5:**
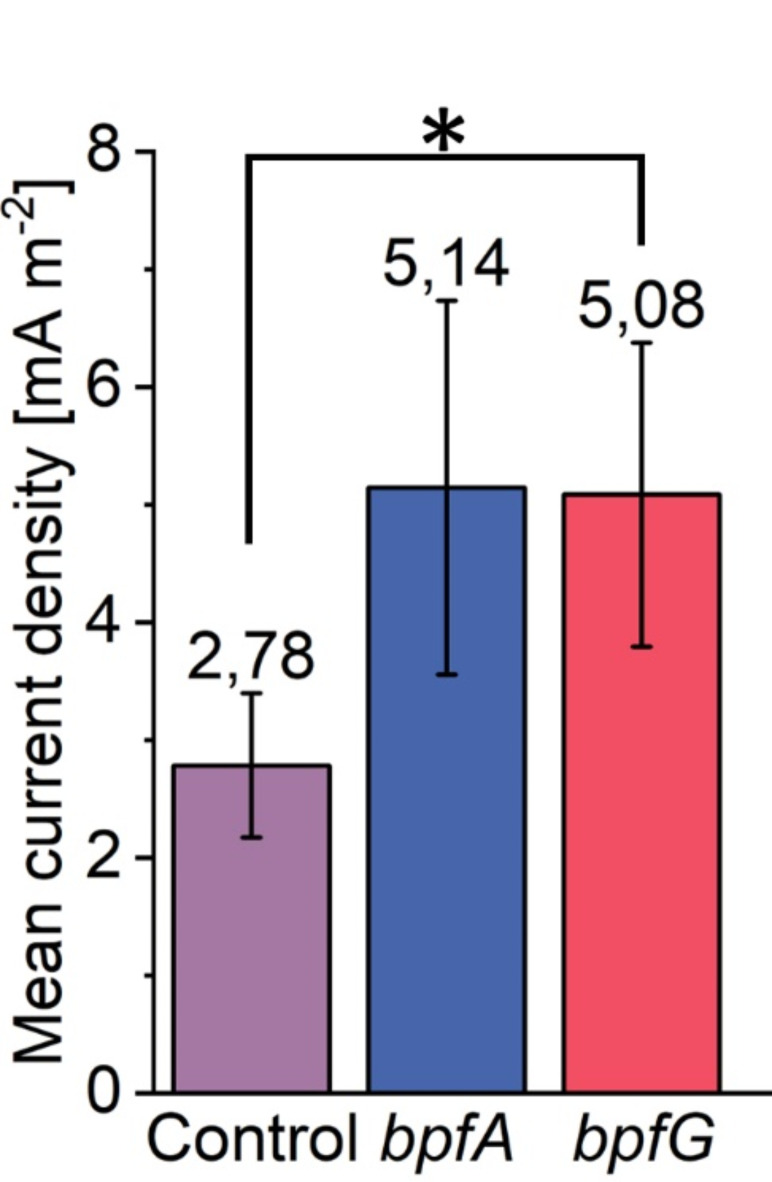
Impact of *bpfG*(C116S) as well as overexpression of *bpfA* on mean current density in microfluidic bioelectrochemical reactors. Error bars represent the standard deviation from individual replicates (*n* = 3). Asterisk represents significant differences to the purple control strain (unpaired t-test: * = *p* < 0.05).

**Fig. 6:**
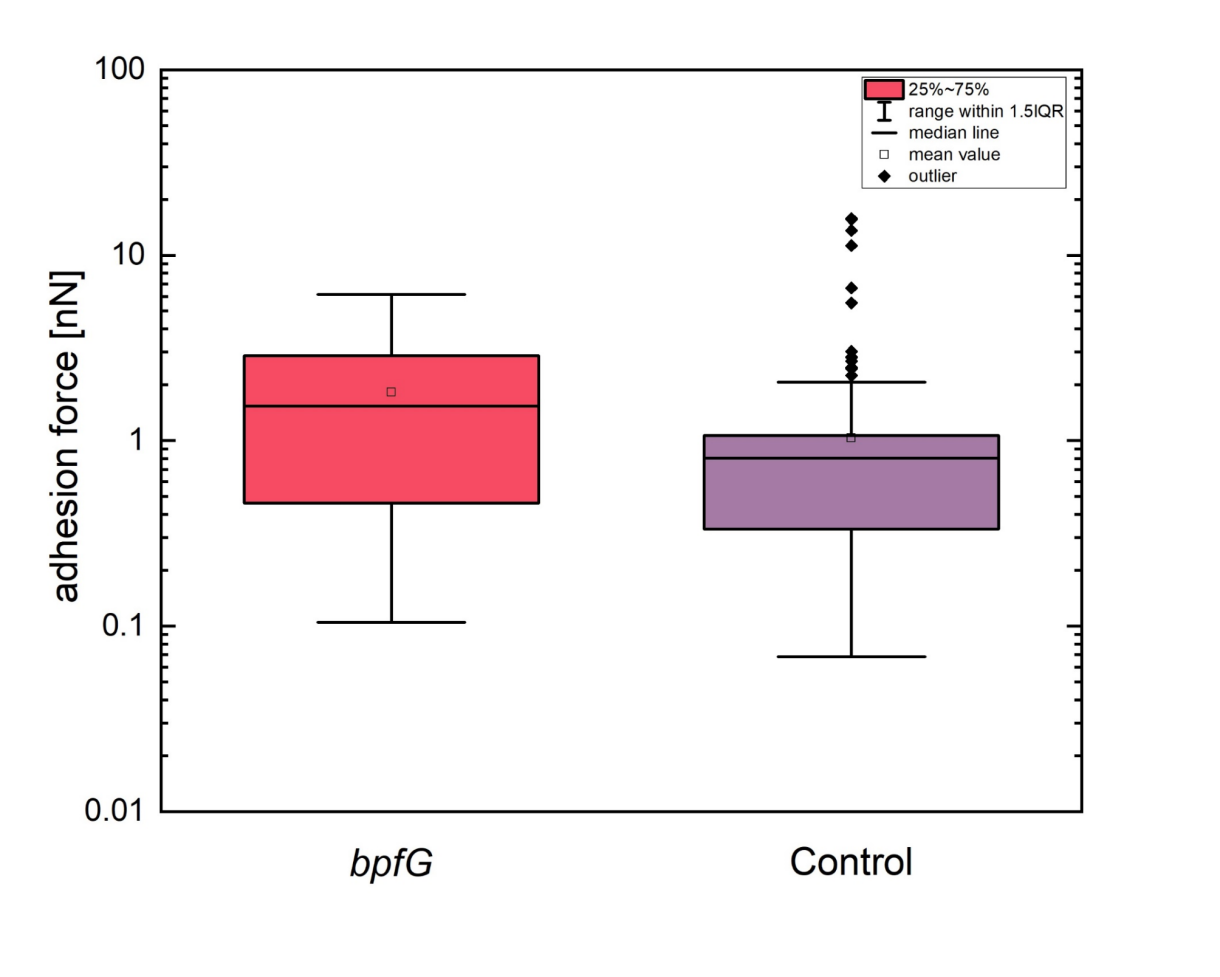
Min-to-max box plots of the adhesion force for two *S. oneidensis* variants on OTS. For the *bpfG* (C116S) mutant five individual cells were measured, for the control four bacterial cells. To obtain a comprehensive overview of the adhesion events, all countable events were summarized for both variants. This yielded 306 evaluable curves for *bpfG* and 286 curves for the control group. The following statistical values are presented for each variant: Mean (open square) and median value (horizontal line), interquartile range (IQR, i.e. the distance between first and third quartile, represented as box height), 1.5* IQR (whiskers) and outliers (black diamonds), i.e. data points that fall outside 1.5*IQR.

The effect of the two mutations on current production was investigated using microfluidic bioelectrochemical flow cells. As shown in Fig. 5, both exhibited a similar positive effect, each resulting in a 1.8fold increase in mean current density. However, it was not possible to collect biofilm data as the ability of *S. oneidensis* to form anodic biofilms is somewhat limited^43–45^ and the biofilm thickness fell within the lowest range of measurement sensitivity for the OCT device (8 × 8 × 5.5 μm). Nevertheless, the results are in line with a recent study by Lin et al.^46^. , which analyzed the impact of different buffer systems on the bioelectrochemical performance of *S. oneidensis* cells. The authors found that current density increased when using PBS instead of PIPES as the buffer system. Transcriptomic analyses revealed that this increased current density correlated with upregulation of *bpfA* (2.8-fold), as well as two other biofilm-promoting factors, *aggA* (2.3-fold) and *csgB* (3.9-fold). Although, we were unable to measure biofilm formation on the electrode surfaces, we aimed to investigate whether activation of the *bpf*-system affected the surface properties of individual cells, potentially enhancing attachment to the electrode surface and leading to higher current densities. Hence, single-cell force spectroscopy experiments were conducted. Due to the complexity of the methodology and as both *bpf*-mutants exhibited the same increase in current density and very similar phenotypes, the experiments were only conducted using the strain expressing *bpfG* (C116S). A hydrophobic surface was used to mimic the hydrophobic graphite electrode material as well as the PDMS surface used as biofilm substratum in the experiments conducted with oxygen or fumarate as electron acceptors. The results from 96 force-distance curves indicated that the *bpfG* mutant had roughly double the adhesion force (mean and median) compared to the control strain expressing the wild-type form of *bpfG* when interacting with the hydrophobic OTS surface (Fig. 6). In general, the adhesion force of the *bpfG* strain on hydrophobic surfaces like PDMS or the graphite anode appeared to be higher, which correlates with improved attachment and increased current density production (Fig. 5), as well as faster surface coverage (Fig. 4) compared to the control strain. It is important to note that enhancing the attachment of microorganisms to hydrophobic surfaces can be achieved not only by increasing their hydrophobicity through genetic manipulation but also by chemically modifying the hydrophobicity of the substratum. For instance, chemical treatment or electrochemical exfoliation can impart hydrophilic to super-hydrophilic properties on graphite electrodes and might thereby influence surface attachment of microorganisms^47–49^.

The *bpfG* (C116S) mutation results in increased levels of BpfA on the cell surface. It is already known that LapA, the homologous protein to BpfA in *Pseudomonas fluorescens*, is essential for initial attachment and biofilm formation on abiotic surfaces, regardless of their hydrophilicity^26,50^. This additional factor might also play a role in explaining the better surface attachment and eventually higher current densities of this strain. The elevated levels of BpfA could also account for the sloughing events seen in the *bpf* mutant strains during aerobic biofilm growth experiments. The increased stability of tfhe biofilm, due to BpfA-mediated cell-cell adhesion, may prevent smaller biofilm clusters from detaching, leading to larger detachment events instead. To guide the deposition of *S. oneidensis* biofilms on electrodes, Zhao et al. utilized a light-inducible synthetic switch to express the autoaggregation proteins CdrAB from *Pseudomonas aeruginosa*, Ag43 from *E. coli*, and AggA from *S. oneidensis*^51^. Expression of CdrAB resulted in measurable autoaggregation, which was later harnessed to direct biofilm formation on transparent indium tin oxide electrodes. Notably, while CdrAB expression enhanced biofilm formation on the electrodes, the measured biofilm thickness remained significantly below the levels observed in this study using fumarate as an electron acceptor, reaching around 10 μm, which is close to the optical coherence tomography (OCT) detection limit, whereas uninduced cells formed biofilms approximately 2 μm thick. Given that Zhao et al. did not perform their experiments under continuous flow, it is likely that biofilms could have been even thinner under such conditions. Nevertheless, although Zhao et al. did not measure continuous current densities, their findings, along with ours, suggest a specific limitation in forming biofilms on anode surfaces.

It is noteworthy that biofilms cultivated under oxic conditions reached heights of 175 to 215 μm after 93 h. Oxygen, as the electron acceptor, must diffuse into the biofilm, and it is reasonable to assume that anoxic conditions prevail beyond a certain biofilm thickness. The penetration depth of oxygen is influenced by several factors, including microbial activity and biofilm density, making it difficult to provide a general estimate. However, it is likely that oxygen is either fully or nearly fully consumed at depths of around 100 μm^52,53^. In contrast to the oxic experiments, all strains formed robust biofilms under anoxic conditions with fumarate as the electron acceptor, with no evidence of detachment during the 50-hour experimental period. This suggests that instability is more likely to occur when biofilms exhibit steeper gradients. There is considerable evidence indicating that oxygen depletion in deeper biofilm layers can lead to disaggregation and detachment^54–56^. In contrast, in the anoxic experiments, the electron acceptor is present in a much higher molarity (100 mM fumarate vs. 252 µM oxygen), which facilitates mass transport into deeper biofilm layers.

## Conclusion

*S. oneidensis* plays a critical role in microbial electrochemical systems. Since cellular adhesion to the electrode surface is essential for these processes, biofilm growth becomes a key feature. *S. oneidensis* plays a critical role in microbial electrochemical systems. Since cellular adhesion to the electrode surface is essential for these processes, biofilm growth becomes a key feature. While biofilm formation by *S. oneidensis* has been extensively studied under oxic conditions^21–25^, little is known about the mechanisms that trigger biofilm formation under anoxic conditions or about the biofilm architecture and composition that could enhance current generation. This study demonstrates for the first time that the limitation on biofilm formation by *S. oneidensis* is not due to the absence of oxygen, but rather to the specific environment of the electrode itself, which seems to restrict the formation of thicker biofilm structures. This implies a need for cell-cell electron transfer, which can occur in *S. oneidensis* through direct interactions among outer membrane cytochromes of individual cells or via outer membrane vesicles that shuttle electrons between cells. Notably, the inhibitory effect of the anode on biofilm formation could not be mitigated by employing *bpf* mutants; however, current production did increase significantly, suggesting improved cell attachment. Interestingly, overproduction of the *bpf*-system had an effect only under anoxic conditions, indicating that different factors may regulate biofilm formation under oxic conditions. Overall, these findings underscore the importance of understanding the differences between aerobic and anaerobic biofilm formation, as they may be governed by distinct regulatory and biochemical mechanisms.

## Supplementary Information

Supplementary Material 1

## Data Availability

The data sets generated and analyzed during this study are shown in the manuscript or can be obtained from the corresponding author on request.
