# Supplementary material for: Comparative analysis of the influence of BpfA and BpfG on biofilm development and current density in Shewanella oneidensis under oxic, fumarate- and anode-respiring conditions
Source: Sci Rep. 2024 Oct 5;14:23174. doi: 10.1038/s41598-024-73474-w (PMC11455927; doi:10.1038/s41598-024-73474-w)
Supplement: Supplementary file 1 — Supplementary Material 1 [file 41598_2024_73474_MOESM1_ESM.pdf]

SUPPLEMENTARY INFORMATION

Supporting and additional information on our results. This file contains images and data to give a better impression of our experiments and findings as well as the results of the accompanying and evaluating experiments.

Journal name: Scientifc Reports

Article title: Comparative analysis of the influence of BpfA and BpfG on biofilm development and current density in *Shewanella oneidensis* under oxic, fumarate- and anode-respiring conditions

Edina Marlen Klein[a], Hannah Heintz[b], René Wurst[a], Simon Schuldt[a], Hendrik Hähl[b], Karin Jacobs[b,c] and Johannes Gescher[a#]

[a] Institute of Technical Microbiology, University of Technology Hamburg, 21073 Hamburg, Germany.

[b] Experimental Physics, Saarland University, Center for Biophysics, 66123 Saarbrücken, Germany.

[c] Max Planck School Matter to Life, 69120 Heidelberg, Germany.

[#] Address correspondence to Prof. Dr. Johannes Gescher, Johannes.gescher@tuhh.de.

ORCiD's:

Edina Marlen Klein: 0000-0001-9642-0218

René Wurst: 0000-0002-6174-7613

Hannah Heintz: 0009-0008-8400-176X

Hendrik Hähl: 0000-0002-2708-0990

Karin Jacobs: 0000-0002-2963-2533

Johannes Gescher: 0000-0002-1625-8810

S1 Strains used in this study.

Strain number	Strain	Source
JG918	*S. oneidensis* MR-1 *Δλ*	Bursac *et al.*[1]
JG1738	*S. oneidensis* MR-1 *Δλ bpfG* (C116S)	This study
JG1739	*S. oneidensis* MR-1 *Δλ bpfA* (P_{cymA})	This study

All strains used in this study can be found in Table S1.

To construct the marker-less mutants of *S. oneidensis*, primers as listed in Table S2 were used.

S2 Primers used in this study.

#	Name	Sequence (5'-3')	Purpose
1	Primer_3474_bpfG_500up_F	AGTGCCAAGCTTGCATGCCTGCAGGTCGAGTGTCATTGAAGCCCG	Gibson ligation: Δ*bpfG*
2	Primer_3475_bpfG_500up_R	TCTGTCTAAACAGTGTCATCTTGGGTGAGCCTACTCTATTGGTGT	
3	Primer_3476_bpfG_500down_F	ACACCAATTAAACACCAATAGAGTAGGCTCACCCAAGATGACACT	
4	Primer_3477_bpfG_500down_R	TACGAATTCGAGCTCGGTACCCGGGGATCGATGGCGACCAAAAAC	
5	Primer_3490_bpfA_PromoterDeletion_500Up_F	AGTGCCAAGCTTGCATGCCTGCAGGTCGAACACCATGGGCTTATG	Gibson ligation: Δ*bpfA*
6	Primer_3491_bpfA_PromoterDeletion_500Up_R	TAATTACCGATCCCATTAAAGAAACATATTTATAACAAATTATTC	
7	Primer_3492_bpfA_PromoterDeletion_500Down_F	ATAATTTGTTATAAATATGTTTCTTTAATGGGATCGGTAATTACA	
8	Primer_3493_bpfA_PromoterDeletion_500Down_R	AGCTCGGTACCCGGGGATCGTATTGCTGTCGTTGGCTAGTATAGAAGGTG	
9	Primer_3502_bpfGC116S_R	TGAATCTCCACCATTGAC	Point mutation *bpfG* (C116S)
10	Primer_3503_bpfGC116S_F2	TCAGAAGATTTTTCAATCGC	
11	Primer_3494_bpfA500Up_CymAP_R	AATGATTAAGTACCACCTTAATAAAGAAACATATTTATAACAAATTATTC	Gibson ligation: P_{cymA}
12	Primer_3495_CymAPromoter_F	TTTGTTATAAATATGTTTCTTTATTAAGGTGGTACTTAATCATTT	
13	Primer_3496_500Down_CymAP_F	TTTGACAATATTTTTGGAGATAGAGTAATGGGATCGGTAATTACA	
14	Primer_3497_CymAPromoter_R	TTGATGTAATTACCGATCCCATTACTCTATCTCCAAAAATATTGT	
15	Primer_3498_bpfAP_Deletion_Test_R	TCGCTATCATTGTTGAGC	Test primers: ΔP_{bpfA}
16	Primer_3499_bpfAP_Deletion_Test_F	TACAAACAGCGAGTCTC	
17	Primer_3500_bpfG_Deletion_Test_F	TCAATATTGACGAGATGACC	Test primers: Δ*bpfG*
18	Primer_3501_bpfG_Deletion_Test_R	CATGTTCAGATAGGGCA	
19	Primer_3504_bpfG_C116S_Test_F	TCGATGACATAAAATTATGGG	Test primers: *bpfG* (C116S)
20	Primer_3505_bpfG_C116S_Test_R	GTGATCCGCATCTTATCT	
21	Primer_0041_pMQ150for	CTGGCGAAAGGGGGATGTG	Test primers: pMQ test
22	Primer_0042_pMQ150rev	CATTAGGCACCCCAGGCTTTAC	

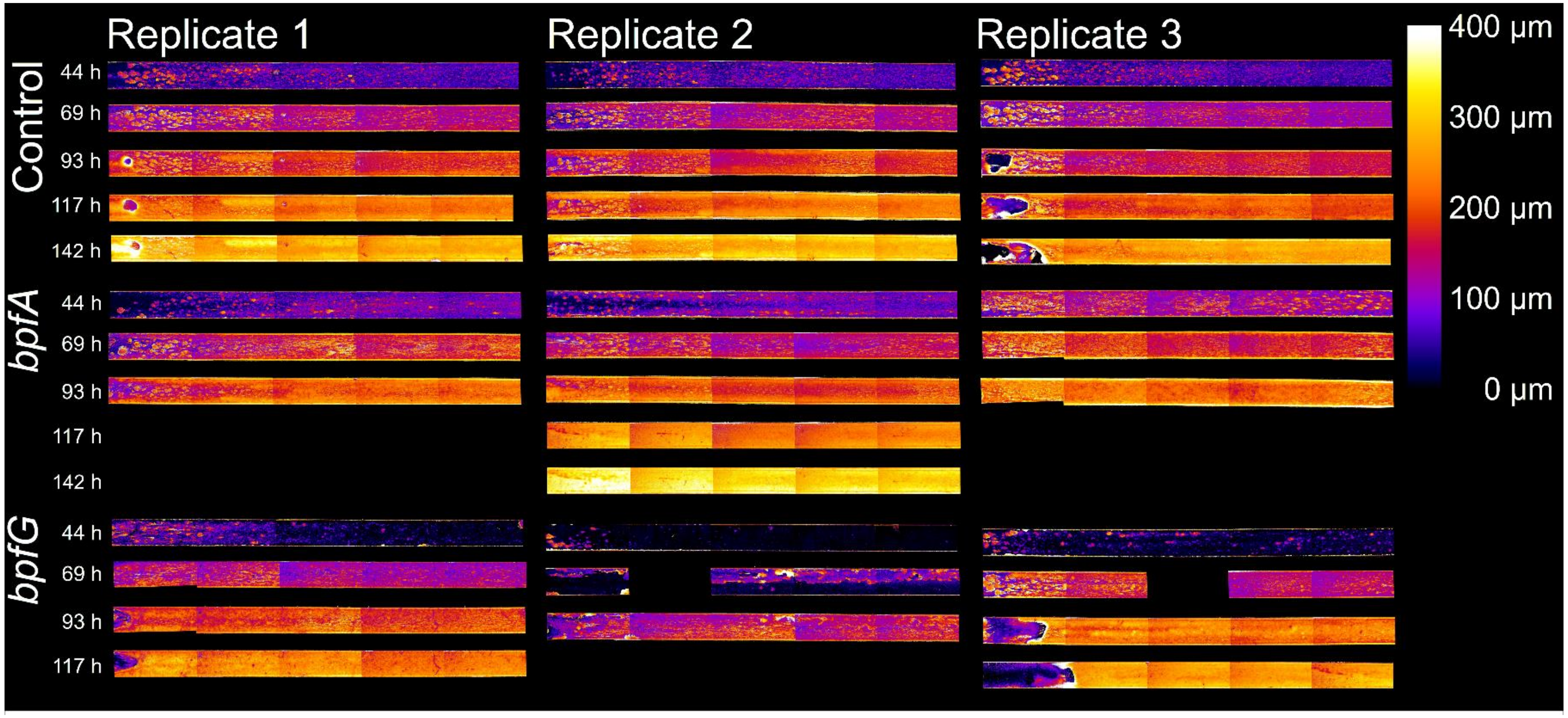

S3 Height maps of biofilms that were cultivated using microfluidic continuous flow reactors with lactate as electron donor. Cultivation was carried out under oxic conditions and the retention time was 5.23 min at a flow rate of 4 mL h^{-1}. After 44, 69, 93, 117 and 142 h five images were taken of each cultivation channel, converted into color-coded height maps by processing and combined into one image by stitching. The cultivation channels shown have a size of 3 x 45 mm and correspond to a top view of the biofilm, with a flow direction from left to right.

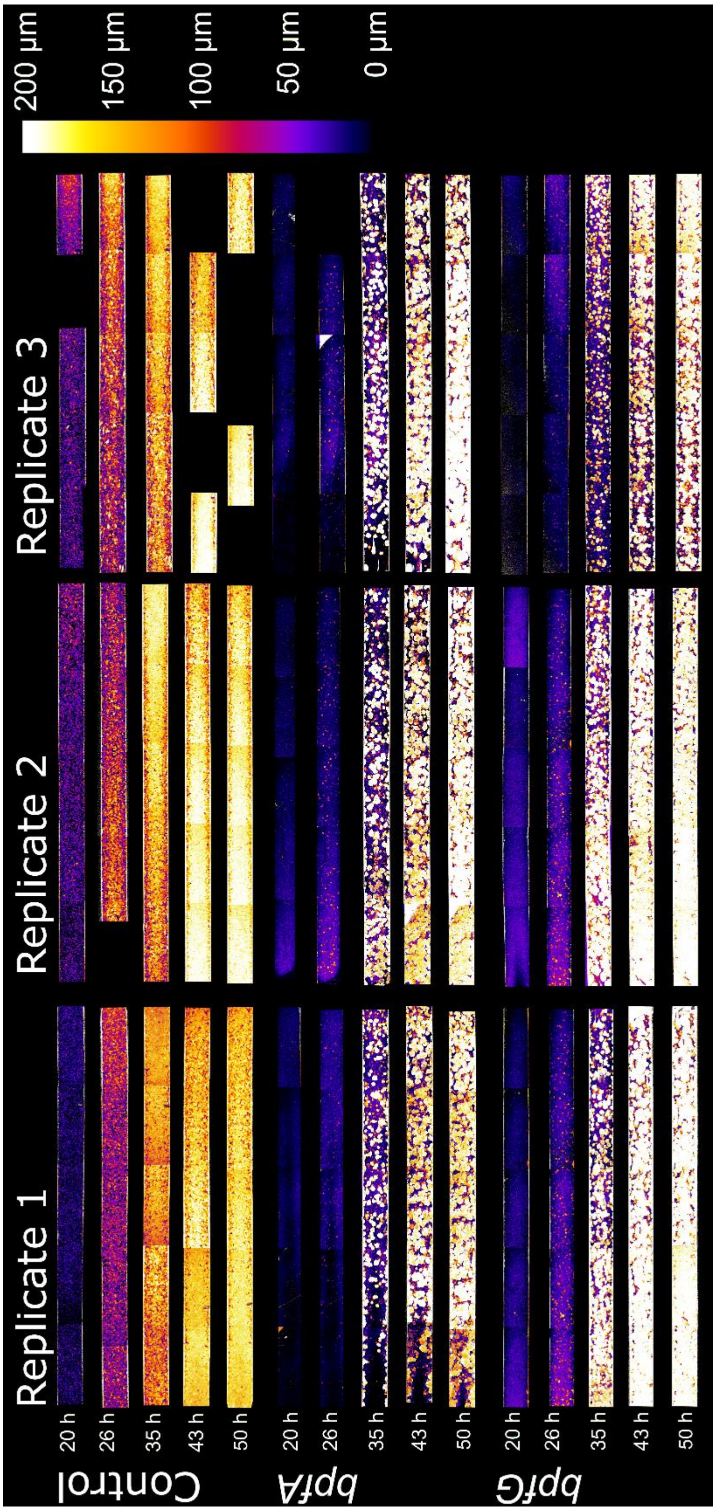

S4 Height maps of biofilms that were cultivated using microfluidic continuous flow reactors with lactate as electron donor and fumarate as electron acceptor. Cultivation was carried out under anoxic conditions and the retention time was 5.23 min at a flow rate of 4 mL h^{-1}. After 20, 26, 35, 42 and 50 h five images were taken of each cultivation channel, converted into color-coded height maps by processing and combined into one image by stitching. The cultivation channels shown have a size of 3 x 45 mm and correspond to a top view of the biofilm, with a flow direction from left to right.

Biofilms were studied using a newly developed microfluidic cultivation platform operating under laminar flow conditions [2].To enable (semi-)automated OCT imaging, a gantry robot (DLE-RG-0003, igus® GmbH, Cologne, Germany) was used to capture images. Development of the biofilm could be monitored throughout the experiment with high reproducibility and low workload. OCT images were acquired with a GanymedeTM spectral domain system (GAN611C1-SP1, Thorlabs GmbH, Dachau, Germany) and analyzed. To describe and quantify the biofilms, the acquired datasets were processed in several consecutive steps. Tables S5-7 contain the mean values and standard deviations of Figure 2. Tables S8-10 contain the mean values and standard deviations of Figure 4. Furthermore,

significant differences to the control strain are marked by colored boxes. Additionally, Figure S3 shows height maps of the biofilms over time as color-coded maps for all three replicates of Figure 1. Figure S4 shows height maps of the biofilms over time as color-coded maps for all three replicates of Figure 3.

S5 Impact of *bpfG* (C116S) as well as overexpression of *bpfA* on biovolume. Cultivation was conducted in microfluidic flow cells under oxic conditions with lactate as electron donor and atmospheric oxygen as electron acceptor. Biovolume in $mm^3\ cm^{-2}$ is given over time. Mean values and standard deviations were calculated from individual replicates (n = 3). Significant differences of the *bpfA* and *bpfG* mutants were calculated in comparison to JG918. Colored boxes indicate significant differences to the control strain (unpaired t-tests: green = $p < 0.01$). An asterisk (*) indicates, that there were two reactors remaining and ** indicates that only one reactor remained.

		44.5 h	70.3 h	93.1 h	117.1 h	143.2 h
JG918	Δλ	6,0 ± 0,3	11,3 ± 0,5	15,4 ± 1,1	19,4 ± 1,5	33,0 ± 2,7
JG1739	Δλ *bpfA* (P_{cymA})	7,4 ± 2,5	13,2 ± 2,3	19,1 ± 1,4	28,8**	39,7**
JG1738	Δλ *bpfG* (C116S)	1,8 ± 0,8	9,2 ± 3,8	15,8 ± 3,2	18,5* ± 0,9	

S6 Impact of *bpfG* (C116S) as well as overexpression of *bpfA* on biofilm height. Cultivation was conducted in microfluidic flow cells under oxic conditions with lactate as electron donor and atmospheric oxygen as electron acceptor. Biovolume in µm is given over time. Mean values and standard deviations were calculated from individual replicates (n = 3). Significant differences of the *bpfA* and *bpfG* mutants were calculated in comparison to JG918. Colored boxes indicate significant differences to the control strain (unpaired t-tests: green = $p < 0.01$; red = $p < 0.05$). An asterisk (*) indicates, that there were two reactors remaining and ** indicates that only one reactor remained.

		44.5 h	70.3 h	93.1 h	117.1 h	143.2 h
JG918	Δλ	79,7 ± 5,4	145,6 ± 5,1	175,4 ± 10,6	211,2 ± 14,4	290,7 ± 16,8
JG1739	Δλ *bpfA* (P_{cymA})	96,1 ± 41,4	168,0 ± 15,7	214,6 ± 8,6	261,2**	345,8**
JG1738	Δλ *bpfG* (C116S)	27,0 ± 14,3	124,8 ± 37,7	185,1 ± 27,7	197,7* ± 28,7	

S7 Impact of *bpfG* (C116S) as well as overexpression of *bpfA* on biofilm porosity. Cultivation was conducted in microfluidic flow cells under oxic conditions with lactate as electron donor and atmospheric oxygen as electron acceptor. Biofilm porosity in % is given over time. Mean values and standard deviations were calculated from individual replicates (n = 3). Significant differences of the *bpfA* and *bpfG* mutants were calculated in comparison to JG918. An asterisk (*) indicates, that there were two reactors remaining and ** indicates that only one reactor remained.

		44.5 h	70.3 h	93.1 h	117.1 h	143.2 h
JG918	Δλ	23,9 ± 1,5	21,7 ± 0,8	12,4 ± 1,2	8,4 ± 1,2	11,7 ± 16,6
JG1739	Δλ *bpfA* (P_{cymA})	28,4 ± 2,4	22,1 ± 8,9	11,5* ± 5,3		
JG1738	Δλ *bpfG* (C116S)	23,8 ± 7,9	29,5 ± 12,8	16,1 ± 5,2	18,9* ± 4,4	

S8 Impact of *bpfG* (C116S) as well as overexpression of *bpfA* on biofilm height. Cultivation was conducted in microfluidic flow cells under anoxic conditions with lactate as electron donor and fumarate as electron acceptor. Biovolume in µm is given over time. Mean values and standard deviations were calculated from individual replicates (n = 3). Significant differences of the *bpfA* and *bpfG* mutants were calculated in comparison to JG918. Colored boxes indicate significant differences to the control strain (unpaired t-tests: green = $p < 0.01$; red = $p < 0.05$).

		**18.17 h**	**23.7 h**	**33.4 h**	**40.7 h**	**47.6 h**
JG918	Δλ	27.3 ± 9.9	81.1 ± 13.9	141.7 ± 10.4	175.0 ± 14.5	180.3 ± 14.6
JG1739	Δλ *bpfA* (P_{cymA})	10.2 ± 2.7	18.8 ± 4.5	114.3 ± 24.3	161.4 ± 19.3	185.6 ± 27.1
JG1738	Δλ *bpfG* (C116S)	17.6 ± 8.6	31.4 ± 10.7	199.4 ± 16.0	236.9 ± 43.6	314.9 ± 78.6

S9 Impact of *bpfG* (C116S) as well as overexpression of *bpfA* on biovolume. Cultivation was conducted in microfluidic flow cells under anoxic conditions with lactate as electron donor and fumarate as electron acceptor. Biovolume in $mm^3\ cm^{-2}$ is given over time. Mean values and standard deviations were calculated from individual replicates (n = 3). Significant differences of the *bpfA* and *bpfG* mutants were calculated in comparison to JG918. Colored boxes indicate significant differences to the control strain (unpaired t-tests: green = $p < 0.01$; red = $p < 0.05$).

		**18.17 h**	**23.7 h**	**33.4 h**	**40.7 h**	**47.6 h**
JG918	Δλ	2.4 ± 0.9	7.6 ± 1.8	11.5 ± 1.2	15.5 ± 1.4	16.1 ± 1.4
JG1739	Δλ *bpfA* (P_{cymA})	1.0 ± 0.2	1.7 ± 0.4	7.2 ± 1.2	9.5 ± 0.9	11.8 ± 1.3
JG1738	Δλ *bpfG* (C116S)	1.8 ± 0.6	3.4 ± 0.4	21.1 ± 12.0	22.2 ± 4.3	22.6 ± 4.7

S10 Impact of *bpfG* (C116S) as well as overexpression of *bpfA* on biofilm porosity. Cultivation was conducted in microfluidic flow cells under anoxic conditions with lactate as electron donor and fumarate as electron acceptor. Biofilm porosity in % is given over time. Mean values and standard deviations were calculated from individual replicates (n = 3). Significant differences of the *bpfA* and *bpfG* mutants were calculated in comparison to JG918. Colored boxes indicate significant differences to the control strain (unpaired t-tests: green = $p < 0.01$; red = $p < 0.05$).

		18.17 h	23.7 h	33.4 h	40.7 h	47.6 h
JG918	Δλ	11.1 ± 2.7	7.8 ± 8.1	19.2 ± 3.8	11.9 ± 0.9	10.9 ± 0.4
JG1739	Δλ *bpfA* (P_{cymA})	8.6 ± 0.4	6.9 ± 3.1	35.3 ± 6.2	41.0 ± 2.7	24.3 ± 17.3
JG1738	Δλ *bpfG* (C116S)	6.6 ± 0.1	7.7 ± 2.0	41.0 ± 0.0	27.5 ± 3.0	26.4 ± 1.1

References

1. Bursac, T., Gralnick, J. A. & Gescher, J. Acetoin production via unbalanced fermentation in *Shewanella oneidensis*. *Biotechnol Bioeng* **114**, 1283–1289 (2017).

2. Klein, E. M., Wurst, R., Rehnlund, D. & Gescher, J. Elucidating the Development of Cooperative Anode-Biofilm-Structures. *Biofilm* **7**, 100193 (2024).
